# Contribution to the Study of Mallein

**Published:** 1892-03

**Authors:** W. Dieckerhoff, R. Lothes

**Affiliations:** Royal Veterinary School, Berlin; Royal Veterinary School, Berlin


### CONTRIBUTION TO THE STUDY OF MALLEIN.

PROF. DR. W. DIECKERHOFF and DR. R. LOTHES, Royal Veterinary School, Berlin. Translated by Leonard Pearson, B. S., V. M. D., Veterinary Department, University of Pennsylvania.

The experiments reported in the last number of the JOURNAL were continued, on both healthy and glanderous horses, and the results obtained coincided fully with those previously reported.

Horses No.'s 15 to 20, inclusive, were injected with Mallein, in 0.5 grm. doses, did not react and the autopsies showed them to be free from glanders.

Horses No.'s 21 and 22 showed symptoms strongly suspicious of glanders, and gave febrile reactions after the injections of Mallein, accompanied by an increased intensity of the symptoms. The discharges from the nose became more profuse, ulcers formed in the nose, abscesses formed in the skin and, in one case, pneumonia developed. The autopsies confirmed the diagnoses of glanders in each case. The article ends with the statement that the experiments made confirm those previously reported by other investigators and proves the specific action of Mallein on glanderous nodules The authors are at present engaged on other experiments of the same character, and promise to report theirr esults as soon as they are complete.

Berliner Thierærztliche Wochenschrift, Vol. 7, No. 51.

